# The Role of p53 in Nanoparticle-Based Therapy for Cancer

**DOI:** 10.3390/cells12242803

**Published:** 2023-12-08

**Authors:** Olga Szewczyk-Roszczenko, Nikolai A. Barlev

**Affiliations:** 1 Department of Synthesis and Technology of Drugs, Medical University of Bialystok, Kilinskiego 1, 15-089 Bialystok, Poland; 2 Department of Biomedicine, School of Medicine, Nazarbayev University, Kerey and Zhanibek Khans St., Astana 020000, Kazakhstan; 3 Institute of Biomedical Chemistry, 10 Pogodinskaya St., Moscow 119121, Russia; 4 Institute of Cytology, 4 Tikhoretsky Ave., Saint-Petersburg 194064, Russia

**Keywords:** p53, gene therapy, nanoparticles, bystander effect, apoptosis

## Abstract

p53 is arguably one of the most important tumor suppressor genes in humans. Due to the paramount importance of p53 in the onset of cell cycle arrest and apoptosis, the p53 gene is found either silenced or mutated in the vast majority of cancers. Furthermore, activated wild-type p53 exhibits a strong bystander effect, thereby activating apoptosis in surrounding cells without being physically present there. For these reasons, p53-targeted therapy that is designed to restore the function of wild-type p53 in cancer cells seems to be a very appealing therapeutic approach. Systemic delivery of p53-coding DNA or RNA using nanoparticles proved to be feasible both in vitro and in vivo. In fact, one p53-based therapeutic (gendicine) is currently approved for commercial use in China. However, the broad use of p53-based therapy in p53-inactivated cancers is severely restricted by its inadequate efficacy. This review highlights the current state-of-the-art in this area of biomedical research and also discusses novel approaches that may help overcome the shortcomings of p53-targeting nanomedicine.

## 1. Introduction

To function properly, multicellular organisms require an exquisitely organized system of quality control. Both endocrine and paracrine regulatory mechanisms define the fate of cells thereby achieving their synchronous propagation or apoptosis. The pivotal element in the system of detection and elimination of defective cells from an organism is the product of the TP53 tumor suppressor gene [1]. The p53 protein belongs to the family of p53 proteins that involve two other members, p73 and p63. Although all members of the family are bona fide tumor suppressors, p73 and p63 play more important roles in the development of multicellular organisms than in oncogenesis [2].

**Figure 1:**
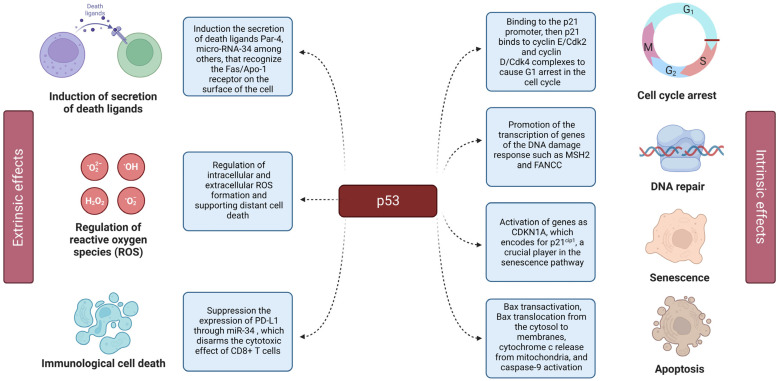
Extrinsic and intrinsic effects of p53. The left panel displays the extrinsic effects of p53, including its by-stander effect. The right panel shows the details of p53 action on the intracellular level (intrinsic effects), including cell cycle arrest, senescence, and apoptosis.

Being a transcriptional factor, p53 promotes the expression of a number of genes involved in the activation of cell cycle arrest and apoptosis [3,4]. In addition, p53 is also able to suppress the transcription of certain genes by augmenting the expression of its target non-coding genes (lncRNAs and microRNAs) [5,6,7] (Figure 1). 

To avoid unnecessary activation of cell cycle arrest and cell death under normal conditions, the intracellular level of p53 is kept in check by post-translational modifications, among which ubiquitinoylation plays a critical role [8,9]. The principal p53-specific E3 ubiquitin ligase is Mdm2 (HDM2 in humans) [10,11], which targets the p53 lysine residues located in the C-terminus and targets the protein for degradation in proteasomes [12]. Importantly, Mdm2 can attenuate the activity of p53 both directly, via the binding and ubiquitylating of the latter and, indirectly, by affecting the interacting partners of p53 [11,13] or the degradation machinery [14,15]. In addition to ubiquitinoylation, other covalent post-translational modifications including the neddylation, sumoylation, and methylation of certain lysins promote p53 inactivation or its proteasomal degradation [16,17,18].

However, when cells experience virtually any type of stress, the signaling cues activate p53 via a sequential attachment of posttranscriptional modifications that include phosphorylation, methylation, and acetylation [19]. Contrary to ubiquitinoylation, acetylation and methylation generally promote p53 stabilization by outcompeting ubiquitinoylation of the same lysins [8]. Moreover, there is a crosstalk between post-translational modifications, and they can regulate each other’s functions in a positive or negative manner [20].

Another important feature of p53 is its promotion of bystander effects in neighboring cells, forcing the execution of a suicidal program in those cells despite the fact that they have not experienced the harmful consequences of stress themselves [21]. This feature of p53 is particularly important for successful elimination of tumor cells by p53-containing nanoparticles.

Thus, p53 acts as an integrator of signaling cascades directed against almost all forms of cell stress and contributes to the maintenance of higher-order structures—tissues and organs. In doing so, p53 employs several modes of action: as a transcriptional activator [22] and as a scaffold for protein–protein interactions [23]. The latter, however, poses the main threat to antitumor defense mechanisms: p53 mutations not only interfere with the normal functioning of defense mechanisms against malignant transformation, but, on the contrary, can lead to an imbalance in signaling cascades, resulting in the emergence of positive feedback loops and, ultimately, leading to cancer [24]. Based on this, many mutations that occur in the TP53 gene transform it from a tumor suppressor into an oncogene. Indeed, TP53 mutations are observed in more than 50% of human cancers [25]. It is important to note that transcriptionally incompetent mutant forms of p53 fail to induce the expression of Mdm2, thereby indirectly stabilizing mutant p53 at the protein level.

Given the fact that p53 can exist in cancer cells in two mutually opposite forms (wt vs. GOF, respectively), the task of designing an effective p53 therapy becomes very challenging. In general, the aim of successful p53-targeted anticancer therapy should be two-fold: (1) activating the p53 molecule in the event of its wild-type conformation and (2) either neutralizing the mutant form of p53 or restoring its wild-type conformation by specific compounds. Apparently, looked at from this perspective, it seems that degradation of p53 by E3 ligases, in particular Mdm2, seems to be the most promising axis for pharmacological intervention in wild-type p53-expressing tumors. In addition to a number of chemical inhibitors of Mdm2 that have already been developed over the years [26,27,28], several new inhibitors have entered clinical trials showing preliminarily promising results [29,30]. However, it should be noted that, so far, the success of Mdm2 inhibitors in clinical settings has been rather limited. There are a few reasons for this: in addition to robust side effects such as thrombocytopenia [31,32] and gastrointestinal toxicity associated with Mdm2 experimental drugs [30], cancer cells were able to adapt to the prolonged therapy by enhancing the expression of other p53-targeting E3 ligases (Pirh2, WWP1, etc.) [33], higher efflux of Mdm2 inhibitors, and their increased metabolic degradation [11]. 

Collectively, it seems that overexpression of exogenously delivered wild-type p53 in cancer cells, together with the Mdm2 inhibition strategy, may yield a better outcome compared to individual approaches. In this review, we discuss the applicability of nanoparticles as a means of delivery of wild-type p53 to cancer cells and the effects of nanoparticles on cellular signaling.

## 2. p53 in Nanoparticle-Based Gene Therapy for Cancer

Delivering the p53 gene in its wild-type (WT) form to cancer cells via gene therapy is an intriguing approach to restoring p53 activity. Among the delivery approaches is the application of adenoviruses as carriers, which has been used effectively in a formulation called gendicine [34]. However, adenoviruses are not always suitable carriers, despite demonstrating encouraging outcomes in preclinical investigations, thus gene therapy may be limited by the absence of an effective delivery mechanism in the late stages of clinical trials [35]. The use of nanoparticles (NPs), which increase the stability of the given particles and exhibit higher absorption by cancer cells, might offer a method for more effectively delivering the p53 gene [36]. Multiple requirements must be accomplished for a p53 gene delivery system to be effective. The vector should not be immunogenic or toxic, permitting numerous injections if necessary. Because the p53 protein is effective but unstable, persistent gene expression within tumors is required for long-lasting therapeutic benefits. Since transfected cells may impact the tumor’s nontransfected areas, substantial levels of transfection may not be required to slow the growth of the tumor. This is because p53 exhibits strong bystander effects discussed in the previous chapter [37]. There are also attempts to deliver peptide activators of the p53 protein into cells, which may provide an alternative to gene therapy [38]. Ideally, the gene vector should be administered systemically and target both primary and metastatic malignancies with precision [39,40]. 

### 2.1. Liposomal Vectors

DDC is a delivery system that is based on DOTAP, DOPE, and Cholesterol. DOTAP (dioleoyltrimethylamino propane) is a cationic liposome, whereas DOPE (1,2-dioeoyl-3-phosphophatidylethanolamine) and cholesterol diminish fibrinogen, prothrombin, and vitamin K affinity for the lipid surface. DDC effectively transported plasmid DNA into ovarian cancer cells. High levels of p53 WT mRNA and protein expression were detected in OVCAR-3 cells because of transfection with the liposome-complexed p53 gene. Compared to control cells, cancer cells transfected with DDC/p53-EGFP complexes showed significant growth suppression. The apoptotic pathway was reinstated in ovarian cancer cells after wild-type p53 function was restored. The volumes of tumors in nude mice were considerably decreased by more than 60% in comparison to the control group after the inoculation of DDC/p53-EGFP complexes [41]. 

A formulation DP3-p53, was chosen for additional in vitro and in vivo testing after a range of cationic liposome-p53 formulations had their in vitro transfection effectiveness and cytotoxicity assessed. In transgenic mice lacking the p53 gene, the capacity of DP3-p53 to insert the p53 gene in the healthy bronchial epithelium was investigated. In vitro experiments using the H358 a non-small cell lung cancer cell line, which lacks the expression of endogenous p53, showed that DP3-p53 could successfully induce G1 arrest, and apoptosis, and introduce and transcribe the p53 gene in the bronchial epithelium of transgenic mice lacking the p53 gene. In studies using groups of four or five mice each, administration of five intratracheal doses of DP3-p53 (2 µg or 8 µg DNA per dose) on days 4–20 after intratracheal tumor inoculation significantly inhibited lung tumor formation and increased by about twofold the survival of mice carrying H358 or H322 endobronchial tumor cells, in contrast to untreated mice and mice treated with the DP3-empty vector [42].

### 2.2. Polymer NPs

A breast cancer cell line MDA-MB-435S treated with D, L-lactide-co-glycolide (PLGA (PLGA is an FDA-approved biocompatible and biodegradable polymer with a wide range of disintegration times and customizable mechanical properties)) nanoparticles containing p53 WT DNA experienced a persistent antiproliferative impact, whose strength increased with time. Plasmid DNA-containing nanoparticles were created using a multiple emulsion–solvent evaporation process. Researchers tracked the intracellular trafficking of the nanoparticles and the nanoparticle-entrapped DNA. They measured the amounts of p53 mRNA over time to comprehend the mechanism of sustained gene expression with nanoparticles. When compared to cells of the MDA-MB-435S transfected with bare p53 WT DNA or p53 WT DNA complexed with a commercially available transfecting agent (Lipofectamine), cells transfected with p53 WT DNA-loaded nanoparticles showed a persistent and much higher antiproliferative impact. The study’s findings point to the possibility that p53 WT DNA-loaded nanoparticles could be helpful in the treatment of breast cancer [43] and other malignancies linked to p53 gene mutations [44]. 

A dual anticancer delivery system with a PLGA core and a poly (lactic acid) (PLA (PLA is produced from sustainable resources like cornstarch and sugarcane. PLA polymers are compostable and biodegradable)) was created to simultaneously deliver chemotherapy and gene therapy. To deliver chitosan-DNA nanoparticles containing the gene for the p53 tumor suppressor protein (chi-p53) and/or doxorubicin (Dox), loaded in the shell and core phases, respectively, researchers took advantage of the benefits of double-walled microspheres. When Dox was enclosed alone rather than with chi-p53 nanoparticles, its encapsulation effectiveness was much higher. On the other hand, the presence of Dox had no impact on the encapsulation effectiveness of chi-p53 nanoparticles. Chi-p53 nanoparticles were released first as intended, then Dox was delivered simultaneously at a nearly zero-order rate. The release tests were carried out in vitro by moderate heating and agitation of the suspension. As a result, a viable approach was demonstrated that can create double-walled microspheres for encapsulation of gene therapy and chemotherapy drugs [45]. 

### 2.3. Metallic NPs

Because of their scalable design, functional variety, control over particle size and surface, and capacity to distinguish between different types of cells via surface coatings, gold nanoparticles are currently considered to be a highly perspective drug delivery technology. For the delivery of p53 WT to ovarian cancer cells, an EGFR (epidermal growth factor receptor)-targeted method based on gold nanoparticles was created. EGFR is overexpressed on the surface of many malignancies, including up to 90% of ovarian tumors. Thus, for specific targeting, cetuximab (C225), an FDA-approved monoclonal antibody that targets EGFR was used to deliver the p53 coding DNA to ovarian cancer cells. Targeting ovarian malignancies in vitro (SKOV-3 cell line) and in vivo (SKOV-3 xenograft mice) has shown encouraging results using a sophisticated gold nanoconjugate system (Au-C225-p53) including gold nanoparticles, cetuximab, and the pCMVp53 plasmid. Although xenograft mice in this study demonstrated its usefulness, it is still too early to say if this medication delivery system will advance to clinical trials [46].

Inorganic strontium nanoparticles (SNPs) enhance the transport of genetic material to the human and murine mammary cancer cells in vitro and genetic models in vivo. Following intravenous delivery of fluorescent siRNA-loaded SNPs, biodistribution profiles in the brain, liver, spleen, kidneys, lung, and mammary tumor of BALB/c mice were examined at 1, 2, and 4 h. Tumor size measurements were used to examine the impact of exogenous p53 expression and endogenous MAPK silencing after SNP-mediated in vivo delivery of the p53 gene and MAPK siRNA. Mice treated with SNPs harboring either the p53 gene or MAPK siRNA showed less tumor growth than mice treated with the naked gene or siRNA, despite reduced residual accumulation in tumor tissues. Higher siRNA concentration decreased the regressive activity of the cancer tissues less than the p53 gene, which may be the result of unanticipated off-target effects [47]. 

### 2.4. Other NPs

An integrative method based on the production of nanoparticulated carriers in conjunction with the supercoiled (sc) isoform purification of a p53 tumor suppressor expressing plasmid was developed. Under mild conditions, the sc topoisoform is recovered with great purity and structural stability. Furthermore, naked sc pDNA was encased within chitosan nanoparticles by ionotropic gelation to improve protection and transfection efficiency. The technique’s gentle particle production conditions allowed for a high encapsulation efficiency for sc pDNA. Furthermore, in vitro transfection tests showed the restoration of p53 protein expression, and most crucially, sc pDNA transfected cells had the highest levels of p53 expression when compared to other formulations [48]. 

Short amphipathic peptides that combine with mRNA to generate stable, neutral nanoparticles are the foundation of ADGN technology. On 20 different cancer cell lines harboring various types of p53 mutations (null, deletion, nonsense, and missense), ADGN-531 nanoparticles containing full-length p53-mRNA were assessed. On colorectal SW403 (p53-deleted) and osteosarcoma SaOs2 (p53 null) mouse xenografts, the in vivo effectiveness of IV-administered ADGN-531 nanoparticles was assessed. On PARPi resistant SUM-149PT and OVCAR-8 cells as well as on PARPi-sensitive MDA-MB436 cells, sensitivity to veliparib (PARPi) was assessed in vitro after ADGN-531 treatment. A significant number of cancer cells with p53 null or nonsense mutations are significantly slowed down in their growth by ADGN-531 NPs. Researchers showed that the kind and degree of p53 mutation in the cancer cells closely correlates with ADGN-531-mediated p53 function recovery. In p53 null or nonsense mutated cells, ADGN-531 causes a 60% to 80% suppression of cell growth, and in p53 missense mutated cells, a 20% to 50% inhibition. In the SaoS-2 and SW403 xenograft mice models, intravenous injection of ADGN-531 containing wild-type p53-mRNA resulted in a reduction of 90% and 70% of tumor growth, respectively. Treatments with ADGN-531 NPs are well tolerated and do not cause clinical toxicity or an inflammatory reaction [49]. 

The ARF-mimicking MDM2-trapping peptide nanoparticles (Mtrap NPs) which can reassemble, were developed to treat p53-positive tumors. This approach is based on the fact that the alternative reading frame (ARF) protein sequesters away Mdm2 in cytoplasm, thereby protecting p53 from the Mdm2-mediated degradation. The findings on U2OS, A549, SK-BR-3, and H1299 cell lines revealed that Mtrap NPs respond to MDM2 and build a nanofiber structure which traps Mdm2. Thus, Mtra NPs suppress p53-wild-type cancers by stabilizing and activating p53 via inactivation of MDM2. MtrapNPs were utilized to load and distribute arsenic trioxide, a potent ROS inducer and a p53 cyctein-mutation rescuer, for the treatment of p53-mutated tumors. Both orthotopic and metastatic models were used, and they both demonstrated significant therapeutic results. Thus, the MDM2-trap method addresses both p53 suppression and mutations, and hence turns out to be an effective approach for treating p53-mutated cancers [50].

## 3. Impact of NPs on the p53 Protein

Although the use of nanoparticles has unquestionable benefits in terms of more effective medicine delivery, we must acknowledge the risks of using nanoparticles. Nanoparticles are not innocuous to the body on their own, which should inspire researchers to work towards developing a safer and more effective technology (summarized in Table 1). 

## 4. Toxicity of NPs

**Figure 2:**
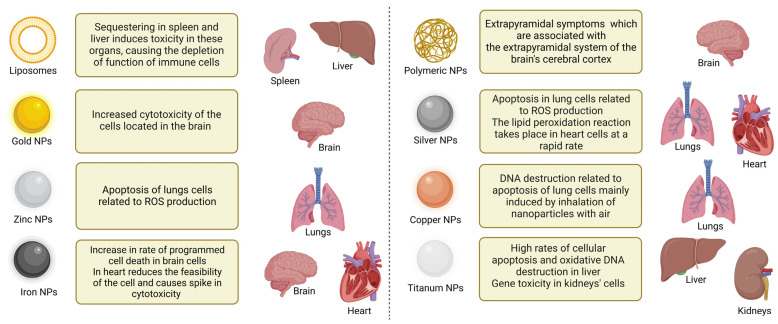
Specific organ toxicity of NPs. Shown are various types of NPs and their effects on different organs.

Metal, lipid, and protein NPs, the three types of NPs commonly utilized in medical delivery, have all been subjected to thorough evaluation of their toxicity profiles (Figure 2). Protein-based NPs, for example, have been linked to hepatotoxicity and nephrotoxicity, whereas metal-based NPs have been linked to increased oxidative stress and may penetrate the cell nucleus [62]. Given the expanding use of NPs in drug delivery, regulatory authorities are rightfully raising concerns about the toxicity of nanocarriers in organisms. Thus, it is critical to identify the gaps in such toxicity studies, including the toxicity of polymeric NPs [63].

In line with this, Adams and colleagues conducted one of the first studies investigating liposomal toxicity in vivo in 1977. Five different types of liposomes with various net surface charges were produced and injected into mouse brains in this investigation. Liposomes containing 45 mol% lecithin or dipalmitoyl lecithin were the least harmful, while others, e.g., containing 9% stearylamine, caused serious cognitive impairment and respiratory failure. These results are important to take into account since liposomes are generally thought to be pharmacologically inactive with low toxicity, yet their safety is highly dependent on the model, exposure period, dose, and/or surface features [64].

The toxicological profiling of the cationic solid lipid nanoparticles (cSLNs) formulation was performed after a single intravenous injection into male Wistar rats at 24 and 72 h. Hematological, biochemical, and histological analyses of the spleen, lungs, liver, and kidneys revealed transient changes, including neutrophilia. The number of macrophages in the lungs, liver, and spleen increased, as did the migration of circulating neutrophils into inflamed tissue concomitantly with the decrease in blood urea nitrogen. Further, cSLNs was detected within the brain parenchyma with no evidence of the blood–brain barrier disruption. These adverse effects proved to be minor and transient (lasting 72 h). These findings emphasize the need to research the toxicity of SLN-based formulations prior to the incorporation of drugs or genes into the formulation and its clinical translation [65].

Silver deposition in the skin and eyes is one of the toxic post-exposure effects in humans. Silver-doped prosthetic restorations color the tissues surrounding the prosthesis blackish blue. After cessation of exposure, these effects are reversible; elimination primarily occurs through the liver and kidney. According to findings generated using the rat liver cell line BRL 3A, the ability of cells cultured with silver nanoparticles was significantly reduced. The researchers discovered a change in mitochondrial membrane potential, a drop in glutathione concentration, and a considerable increase in reactive oxygen species concentration. An in vitro model of alveolar macrophages was also studied, and its vitality fell directly proportionally to the nanoparticle concentration. Increased levels of ROS and pro-inflammatory cytokines such as TNF-, IL-1, and macrophage inflammatory proteins 2 (MIP-2) were found, indicating that oxidative stress is a possible mechanism of action. The researchers investigated the effect of silver nitrate nanoparticles on human periodontal tissue fibroblasts. The size of AgNO_3_ nanoparticles was shown to be inversely related to cell viability. Unlike AgNO_3_ NPs, Ag NPs did not impair the cell viability of periodontal tissue fibroblasts at any of the investigated doses or incubation durations. These findings suggest that silver nitrate nanoparticles with diameters ranging from 10 to 20 nm can cause a limited cytotoxic effect when directly contacted with oral tissue, depending on the dosage and exposure period [66].

The mechanisms that regulate copper metabolism are easily disturbed, resulting in oxidative damage. The most common is a cascade effect that impairs liver function or disrupts mitochondrial respiration. Copper particle accumulation has been reported in plants and animals. The respiratory system is the primary route of toxicity caused by copper nanoparticles. Long-term exposure can result in dose-dependent pneumonia and lung cell damage. CuO NPs induce high toxicity in human lung epithelial cell A549, leukemia cell HL60, human breast cancer cell MCF-7, and hepatocellular carcinoma cell HepG2. Nanoparticles that enter the body through the lungs can also affect the cardiovascular system. One hypothesis is that inhaled particles promote inflammation and release inflammatory cytokines into the bloodstream. Another theory holds that nanoparticles are freely discharged into the circulatory system via lung blood veins. This affects their buildup in specific parts of the blood vessels, directly impacting the cardiovascular system. CuO NPs exhibit genotoxicity as measured by inflammation and oxidative stress activation. Recent research suggests that copper nanoparticle toxicity is caused by lysosome malfunction. This is due to the accumulation of nanoparticles, which causes autophagic stress. CuO nanoparticles are also hazardous to vascular endothelial cells, triggering death in a caspase-independent way [67].

The widespread use of zinc oxide-containing consumables raises alarming health concerns. Sharma et al. discovered significant negative effects on the liver, which is the fundamental site of metabolism in living organisms. Zinc oxide has both genotoxic and apoptotic potential in the HepG2 hepatoblastoma cell line when tested at 14–20 g/mL for 12 h [61].

Many findings suggest various sorts of toxicity, such as genotoxicity of ZnO NPs against BIP/GRP78 cell lines. Furthermore, mutagenicity of these particles against Chinese hamster lung fibroblast cells has also been observed. The use of zinc particles has been linked to neurotoxicity and lung damage. Despite this, several researchers claim that nanoparticles have beneficial effects at lower concentrations; however, these benefits differ depending on the mode of administration, duration of exposure, cell lines, and organs or tissues studied [68].

The toxicity of iron oxide NPs may be increased by oral and inhalation methods of administration rather than injection, according to research. The sensory nerve pathway can induce several unique effects that are not seen with other routes of administration. The neurological system, heart and lungs, thyroid gland, and organs of the mononuclear phagocytic system are among the organ systems that may be harmful when exposed to iron oxide nanoparticles. The reproductive system and the influence of nanoparticles on the health of the first and second generations of humans exposed to the hazardous effects of iron oxide nanoparticles have a unique position. This information should be used in future research on the toxicity of iron oxide nanoparticles [69].

Despite the fact that PEG is assumed to be non-immunogenic, due to a number of physicochemical features, there is growing evidence that PEG generates immunogenic reactions when coupled with other materials such as peptides and nanocarriers. PEG in combination with other elements can result in the development of anti-PEG antibodies after injection under these conditions. The generated antibodies appear to have a negative impact on the therapeutic effectiveness of subsequently injected PEGylated formulations. Furthermore, hypersensitivity to PEGylated compositions could be an important impediment to PEGylated product usability. Several studies have connected the existence of anti-PEG antibodies to cases of complement activation-related pseudoallergy (CARPA) caused by PEGylated formulations [70].

Long-term investigations utilizing high-pitched dosages of biodegradable polymers have revealed limited information regarding their safety. As a result of the reactivity and size reduction in the polymers chosen by using living cells other than the target, these cells can exhibit extrapyramidal symptoms which indicate toxicity to the nervous system. With a better understanding of polymers and their properties, it is also critical to emphasize their safety and toxicity. Toxic effects of polymeric nanodrugs include increased cytotoxicity, decreased cell feasibility, increased rate of apoptosis, precursors for tumor formation, DNA destruction, gene toxicity, cell membrane rupture, and lipid peroxidation reactions [71].

Chitosan, poloxamer 188 (PF68), and poly(vinyl alcohol) (PVA (PVA is utilized in several medical applications due to its biocompatibility, low protein adhesion tendency, and low toxicity)) were used as stabilizers to create either neutral or positively and negatively charged PLGA nanoparticles. All tested nanoparticles showed no or minimal signs of toxicity on human-like THP-1 macrophages when used at therapeutically relevant concentrations (up to 0.1 mg/mL in vitro), as measured by cell mitochondrial activity, induction of apoptosis and necrosis, production of intracellular reactive oxygen species, and secretion of pro-inflammatory cytokines. Cytotoxicity was observed to be generated by the presence of stabilizers at high doses (more than 1 mg/mL), regardless of the toxicological pattern of the stabilizer itself. While stabilizer-free PLGA nanoparticles revealed no cytotoxicity, when used as a nanoparticle stabilizer, the slightly cytotoxic chitosan polymer conferred significant cytotoxicity to PLGA nanoparticles; more surprisingly, the otherwise harmless PVA and PF68 polymers also conferred significant cytotoxicity to PLGA nanoparticles. These findings revealed the critical toxicological contribution of stabilizers used in the formulation of PLGA nanoparticles when used at high concentrations, which may have implications for the local toxicities of PLGA-based nanomedicine, as well as new information on the cytotoxic effects of internalized nanoparticles [72].

## 5. Limitations and Future Perspectives

There are several significant obstacles that must be overcome for p53-based gene therapy to gain clinical relevance. The first problem stems from the nature of mutations in the p53 gene: the most frequent mutations in p53 found in cancer cells exhibit dominant negative effects towards p53 WT, thereby reducing or even completely blunting its tumor suppressive activity. Since GOF mutants of p53 are not degraded by Mdm2 due to their structural features and their inability to induce the Mdm2 expression, they incorporate in cells at high levels and often outcompete the exogenously delivered p53 [73]. Perhaps not surprisingly, it was found that in a population of cancer cells with mixed p53 genotypes, cells bearing p53 WT responded better to p53 therapy compared to those cells that contained mutant p53 [74,75].

One of the solutions to this problem is to re-engineer the p53 molecule by either increasing its expression levels and/or alter its structure to avoid the interaction with mutant p53 [76,77]. For example, chimeric proteins resulting from the fusion of mitochondrial targeting signals (MTS) and functional domains of p53 (e.g., the DNA binding domain) are capable of rapidly (but transiently) inducing apoptosis through their action inside the mitochondria. In this way, the MTS-p53 fusion proteins can escape the interaction with endogenous p53 located in the nucleus and cytoplasm of cancer cells [78]. This is a new mechanism of apoptosis that is not associated with transcriptional activation of p53 target genes in the nucleus. Another variant of the same approach is represented by the p53-Bad chimeric protein, which is a fusion product of monomeric p53 with the proapoptotic factor Bad and contains an integrated MTS [79]. Both chimeric proteins (p53-MTS and p53-Bad) were shown to successfully overcome the dominant negative effect of mutant p53. This new gene therapy approach showed the ability to trigger apoptosis in cancer cell lines with various p53 mutations, indicating that it could be used as a therapy regardless of p53 status.

Other enhanced p53 gene therapies include creation of “super-p53,” in which the p53 tetramerization domain (TD) is replaced with alternative TD. The tetramerization domain is required for effective transcriptional activity of p53. However, at the same time this domain allows wild-type p53 to hetero-oligomerize with dominant negative p53 mutants. To circumvent this undesirable phenomenon, the TD of wild-type p53 was replaced with an engineered leucine zipper that assembles into a four-stranded coiled coil. The ability of the engineered zipper to drive tetramerization was critical to p53 function, since p53 molecules engineered only to dimerize have been shown to be poor tumor suppressors [80].

A similar approach was used in a study by Lim’s group. In this work, they replaced the native tetramerization domain of p53 with a coiled coil from the B-cell receptor (BCR), which itself is a potent inducer of apoptosis. This artificial p53-CC protein became inert to the dominant negative inhibition by mutant p53 due to its inability to tetramerize with mutant p53 [81]. Importantly, p53-CC was still able to tetramerize with itself and activate a large number of p53 target genes, which is critical for robust tumor suppression in vivo [82].

The second major obstacle to p53-based gene therapy relates to limitations of delivery systems, including nonspecific toxicity. On the one hand, the half-life of most NPs delivered systemically is estimated in minutes due to the activity of residential macrophages that phagocyte them. On the other hand, shielding NPs from phagocytosis by layers of negatively charged polymers increases the time of their persistence in the blood stream, yet it diminishes the specificity of their targeting, thereby increasing the level of non-specific toxicity in neighboring tissues. To this end, great promise is held by an approach of antibody-directed NPs that can deliver a DNA payload [46]. It should also be noted that numerous non-viral delivery vehicles, such as liposomes, are constrained in their capacity to provide a controlled release of DNA and may need multiple doses for substantial periods of time to achieve effective therapeutic results. Polymeric NPs may therefore provide a more effective alternative. Since NPs enter cells by interacting or fusing with the plasma membrane, which might result in nonspecific cell lysis, they also have cytotoxicity restrictions [83]. Due to persistent gene expression (requiring less frequent dosage) and the biocompatibility of the polymer system, NPs may consequently have a wider therapeutic window than conventional gene vectors, especially for systemic delivery. In this regard an interesting approach to increase the targeted delivery of NPs was developed using the RGD-targeting peptide. The RGD sequence first identified in fibronectin, is the principal integrin-binding domain present within several ECM proteins including fibronectin, some laminins, and collagens. Since the RGD sequence can bind to multiple integrin species, synthetic RGD peptides conjugated with biodegradable polymers would provide selective advantages for biomaterials applications in terms of specific targeting the cancer-associated ECM [84].

The third reason for the low efficiency of p53-based therapy is the rapid adaptation of cancer cells to p53 overexpression through the mutation of apoptotic pathways, decreased mitochondrial activity, and overexpression of proteins that suppress p53. Caspases represent an instrumental part of the apoptotic process working as effectors for both extrinsic and intrinsic signaling cues [85]. Accordingly, caspase genes are often mutated in different types of cancer. For example, several types of tumor were reported to gain mutations in the caspase 8 gene, which is critical for both extrinsic and intrinsic apoptosis [86,87]. In addition, caspase-3 is often mutated in breast cancer [88]. Notably, the MCF-7 cell line that is widely used in molecular cancer studies also lacks caspase-3. This deficiency in MCF-7 cells is caused by skipping of the exon 3 during pre-mRNA splicing, thereby resulting in premature abortion of the CASP-3 mRNA translation [89].

Another mechanism of preventing apoptosis is exerted by cancer cells via down-regulation of mitochondria. Although at first counter-intuitive, since mitochondria are required for efficient cell proliferation, this mechanism allows cancer cells to survive because mitochondria produce ATP, which is required for apoptosis. Multiple mechanisms exist to shut down the mitochondria activity in cancer cells. One them is via overexpression of adenylate kinase, AK-4, which in turn, attenuates AMPK, the major cellular switch from anabolism to catabolism in cells [90].

Finally, cancer cells may overcome overexpression of p53 by elevating the production of negative interacting partners of p53, thereby blunting its apoptotic activity [91,92].

To overcome these significant challenges developed by cancer cells in response to p53 therapy, several steps need to be taken. A better design of NPs should allow longer time of circulation of NPs with p53 payload and improved specificity of targeting. Furthermore, multi-carrier NPs allowing the combinatory treatment of cancer cells with p53 mRNA and p53-enhancing drugs hold particular promise [93]. Finally, combination of p53 therapies with immunotherapies seems to be an attractive approach. In fact, successful attempts combining p53 therapy with immune checkpoint inhibitors [94], or T-cell transfer that recognize p53 mutant neoantigens [95] are very encouraging.

From the clinical point of view, the most successful was p53 gene therapy using the intertumoral delivery of the p53 transgene by adenoviruses [96]. In fact, the first recombinant p53 therapeutic drug approved in China, gendicine, is expressed in adenovirus developed by Shenzhen SiBiono GeneTech.

With intratumoral administration of adenoviral particles, a complete local response was observed, as shown by positron emission tomography data [97], and biopsy documented a complete pathological response (in combination with radiation therapy) [98]. Despite these impressive data, the widespread clinical application of this approach has not gained further acceptance for several reasons. First, such intertumoral approaches fail to treat metastatic diseases. Secondly, due to the lack of systemic delivery vehicles it was impossible to effectively target both primary and metastatic sites. In this respect, the most successful formulation of nanoparticle-mediated delivery of the p53 transgene is SGT-53 in combination with gemcitabine/nab-paclitaxel, which is in phase II of clinical trials against colorectal cancer [99] (for review see: [100]).

Importantly, nanoparticles through intravenous administration are more suitable for treating distant metastases than intratumoural injection. Furthermore, the improvement of delivery methods for gene products specifically to cancer cells has greatly increased the efficiency and specificity of nanoparticles, improving their ability to selectively restore p53 expression in cancer cells and enforce more robust anticancer effects [101].

There is also the possibility of a paradoxical approach to p53 therapy, which appears to have been effective in metastatic melanoma. Even in drug-sensitive tumors, eradicating all melanoma cells is ineffective in patients because a percentage of cells might enter a slow-cycling condition, keeping them resistant to many targeted therapies [102,103]. It is yet unknown which processes distinguish these subpopulations and promote the resistant phenotype. Wnt5A promotes a metastatic, chemotherapy-resistant phenotype, stabilizes p53 half-life and exploits p53 to enter a slow-cycling state in response to stress. Inhibiting p53 inhibits the slow-cycling phenotype which makes melanoma cells more sensitive to BRAF/MEK inhibition. This can be performed in vivo with a single dose of p53 inhibitor at the beginning of BRAF/MEK inhibitor therapy [104].

## 6. Conclusions

Gene therapy has the potential to become the treatment solution of the future. The transfer of genes coding, for example p53 protein, to malignant cells is one example of potential cancer therapy. Previous efforts that used adenoviruses as carriers were not always successful, thus the development of new systems that use nanoparticles as carriers appears to be promising. Nanoparticles are not the most perfect carriers and, depending on the physical properties of the fabrication material, have distinct limitations and toxicity to various organs. When developing gene therapy based on the p53 protein, it is critical to determine empirically which nanoparticles have the most efficacy and cause the least harm to the patient.

## Figures and Tables

**Table 1:** Interaction of NPs and p53.

Type of NPs	In Vitro/ In Vivo	Tissue/Cell Line	Effect	Reference
**Al_2_O_3_**	In vivo	Sub-brain regions of rats	Decreased expression of cyclin D1, bcl-2, Mdm2, and phospho-Rb and increased expression of p53, p21, Bax, and Rb	[51]
**Ag**	In vitro	GC1415, NCI-N87, and MKN45	Increased p53 expression, inhibition of STAT3	[52]
	In vitro	HCT116	Increased transcription of p53, p21, and caspases (3,8,9), decreased amount of AKT and NF-κB	[53]
**CuO**	In vitro/ Ex vivo	K562 and peripheral blood mononuclear cell	Increase in Bax/Bcl-2 ratio, upregulation of p53, and ROS production	[54]
**Fe_3_O_4_**	In vitro	HepG2, A549, IMR-90	Induction of ROS, upregulation of p53, and caspases 3 and 9	[55]
**Pt**	In vitro	IMR-90, U251	Upregulation of p53 and p21, DNA damage	[56]
**Si**	In vitro	HUVECs	Activation of c-Jun, p53, caspase-3, and NF-κB, increased Bax expression and suppression Bcl-2	[57]
**TiO_2_**	Ex vivo	peripheral blood lymphocytes	Accumulation of p53 and activation of DNA damage checkpoint kinases	[58]
	In vitro	PC12	ROS and JNK/p53 mediated apoptosis and causing. G2/M arrest by the activation of p53/p21 pathway	[59]
**V_2_O_5_**	In vitro	B16F10, A549, and PANC1	Impaired angiogenesis, increased ROS, overexpression of p53	[60]
**Zn**	In vitro	HepG2	ROS generation, DNA damage, activation of p53 and p38	[61]
